# Mineral nitrogen captured in field-aged biochar is plant-available

**DOI:** 10.1038/s41598-020-70586-x

**Published:** 2020-08-14

**Authors:** Ghulam Haider, Stephen Joseph, Diedrich Steffens, Christoph Müller, Sarasadat Taherymoosavi, David Mitchell, Claudia I. Kammann

**Affiliations:** 1 Department of Plant Biotechnology, Atta-Ur-Rahman School of Applied Biosciences, National University of Sciences and Technology, (NUST) Campus, H-12, Islamabad, Pakistan; 2 School of Materials Science and Engineering, University of NSW, Kensington, NSW 2052 Australia; 3 Discipline of Chemistry, University of Newcastle, Callaghan, NSW 2308 Australia; 4 Department of Plant Nutrition, Justus-Liebig University Giessen, Heinrich-Buff Ring 26-32, 35392 Giessen, Germany; 5 Institute of Plant Ecology, Justus-Liebig University Giessen, Heinrich-Buff Ring 26-32, 35392 Giessen, Germany; 6 School of Biology and Environmental Science and Earth Institute, University College Dublin, Belfield, Dublin 4, Ireland; 7 Electron Microscopy Centre, AIIM Building, Innovation Campus, University of Wollongong, Squires Way, North Wollongong, NSW 2517 Australia; 8 Climate Change Research for Special Crops, Department of Applied Ecology, Hochschule Geisenheim University, Von-Lade Str. 1, 65366 Geisenheim, Germany

**Keywords:** Plant ecology, Climate sciences, Ecology, Environmental sciences, Solid Earth sciences

## Abstract

Biochar may serve as a tool to sustainably mitigate climate change via carbon sequestration and by improving soil fertility. Biochar has shown to retain nitrate in its pores, which increases with an organic coating of the inner surfaces and residence time in soil (“aging”). Here we investigated the plant accessibility of the captured nitrate in field-aged biochar, as a pre-requisite for developing carbon-based N fertilization techniques with environmental benefits. Based on previous results, we hypothesized that part of the biochar-captured nitrate would remain unavailable for plants. A two-factorial greenhouse experiment was designed, where the N was applied either as Ca(NO_3_)_2_ or as N captured in field-aged biochar at five increasing N doses to quinoa and perennial ryegrass in pots. Interestingly, the biochar-captured N was as plant available as the mineral nitrate, except for the highest dosage. Refuting our hypothesis, no significant amounts of N were extractable at the end of the study from the biochar–soil mixtures with repeated-extraction protocols. Thus, N captured by biochar may improve the N use efficiency in agriculture. Further research should evaluate the role of biochar particle size, root morphology, mycorrhization, and soil moisture (variations) for nitrate retrieval from biochar particles by plants because the captured biochar N was less available in the field as under present controlled conditions.

## Introduction

Since preindustrial times (ca. 1,750), anthropogenic CO_2_ emissions have increased atmospheric CO_2_ mixing ratios by approximately 150% to more than 410 ppm to date, at an unprecedented rate of increase of currently 2.24 ppm per year^1^. In December 2015, the COP21 stated in the Paris Agreement that humanity intends ‘to keep atmospheric temperature rise to well below the 1.5 °C by 2050’. To achieve this goal, it is not only necessary to curb anthropogenic CO_2_ emissions drastically within the next 30 years but also to additionally remove about 300 Gt of carbon from the atmosphere until 2,100 (using negative emission technologies, NETs, e.g.^2,3^; see also www.4per1000.org). Among various NETs, natural climate solutions (NCS) such as afforestation/reforestation, combustion/pyrolysis^4,5^ and soil C sequestration (SOC stock increase; biochar) offer multiple co-benefits, supporting the UN’s sustainable development goals (SDGs^3^). NCS involving soil management strategies play an important role among the available spectrum of NETs. It is estimated that if the full potential of carbon sequestration in the biosphere (vegetation and soil 155 and 178 Pg respectively) is accomplished^6^, it will reduce the atmospheric CO_2_ concentration by more than 150 ppm^7^. For a soil SOC increase, recommended soil carbon sequestration strategies have to be adopted between 2020 and 2100^6^.

Biochar is a solid by-product of pyrolysis, the thermal conversion of biomass at temperatures of 350–900 °C at low to absent oxygen conditions. It has been proposed as a carbon sequestration strategy with environmental co-benefits when used in soils^8–12^ and is considered to be one of the potential NETs in the IPCC 1.5 °C special report published in 2018^2,13^.

Recently, a global meta-analysis of 105 studies revealed that the use of pure biochar, applied in large doses of ≥ 10 t ha^−1^ to soils, resulted in no yield increases in temperate latitudes and an average 25% yield increase in the subtropics and tropics^14^. Another meta-analysis of 153 peer-reviewed studies reported that crop plant productivity is dependent on the consortium of biochar and soil properties^15^. However, using biochar at ≥ 10 t ha^−1^ is economically challenging since the yield increase does not necessarily repay the investment, as long as services to the commons such as N_2_O emission reductions, reduced nitrate leaching^16,17^ or C sequestration^3^ are not paid for by society. Hence, strategies for achieving agronomic benefits need to be developed that allow using lower cost-effective dosages, e.g. by blending biochar with organic fertilizers and applying it at very low rates (0.5–2 t ha^−1^)^18–20^; or by reducing biochar production costs by using local residue biomass and simple, clean techniques^21^. As an example, rice husk biochar treated with urea-H_2_O_2_ was applied as a slow N release fertilizer (17.63% slower release than ordinary fertilizer) in a pot experiment with cabbage, with the additional benefit of cadmium immobilization, resulting in significant yield improvements^22^. A successful commercial strategy has been to create a compound fertilizer that comprises 20% biochar, 5% clay, and 75% NPK with application rates of the biochar at approximately 100 kg ha^−1^^23,24^.

Kammann et al*.*^25^ observed that co-composted biochar particles improved plant growth to a much greater degree compared to pure biochar, since they were loaded with nutrients, in particular nitrate, which was surprising because biochar does not have a large anion exchange capacity^26^. Subsequently, Haider et al.^27^ demonstrated that the nitrate entrapped in field-aged or co-composted biochar particles was partly un-exchangeable by standard extraction methods (e.g. only 13% extracted from a field aged biochar with the first extraction, compared to the total extracted with 10 repeated extractions); in the field, this caused significant nitrate retention in the top soil^28^, a finding underlined by results of a recent meta-study^16^. Joseph et al*.*^29^ found that capture of nitrates after composting followed a series of complex reactions that involved, movement into the pores, then surface adsorption and incorporation into an organomineral layer. Moreover, Hagemann et al*.*^30^ showed that co-composting enriched biochars with nitrate (the magnitude depending on the biochar type) and that the observed nitrate capture was related to the formation of an organic coating on biochar surfaces^31^ as well as the formation of organo-mineral complexes^29^. This is often termed “aging” and does not necessarily mean that the biochar-C itself was oxidized, but rather that the biochar acquired/sorbed non-biochar (aromatic) dissolved carbon and nitrogen species^32^.

Our field experimental results showed significant nitrate retention in biochar-amended sandy topsoil over time^27,28^. The study also showed that there is a slow release of nitrate from picked biochar particles from the field site (with repeated KCl extractions, Haider et al.^27^) where no N fertilizer was applied to one crop (summer barley (*Hordeum vulgare* L.) over the vegetation period in 2014. The hypothesis was that the biochar-amended, nitrate-enriched plots should show higher biomass yields as have higher mineral N in the 0–90 cm soil profile. The results obtained in the unusually hot summer 2014, however, refuted this hypothesis; no yield increase occurred compared to the control without biochar^28^.

These findings instigated the current study. We hypothesized that part of the nitrate captured in the biochar particles would not be plant-accessible and assumed that the biochar would retain a certain amount of N, which would subsequently be unavailable for plant N uptake; and that this feature may be the cause for the sometimes slightly negative effects that pure, production-fresh biochar use can have on crop yields in temperate soils^14^. Hence, we picked naturally nitrate-loaded biochar particles from the field experimental site and used them as nitrate (carbon) fertilizer, comparing biochar-captured nitrate to the same amount of calcium nitrate in a fully randomized pot study with two different plant species under controlled conditions in the greenhouse.

## Results

### Plant biomass production

**Figure 1:**
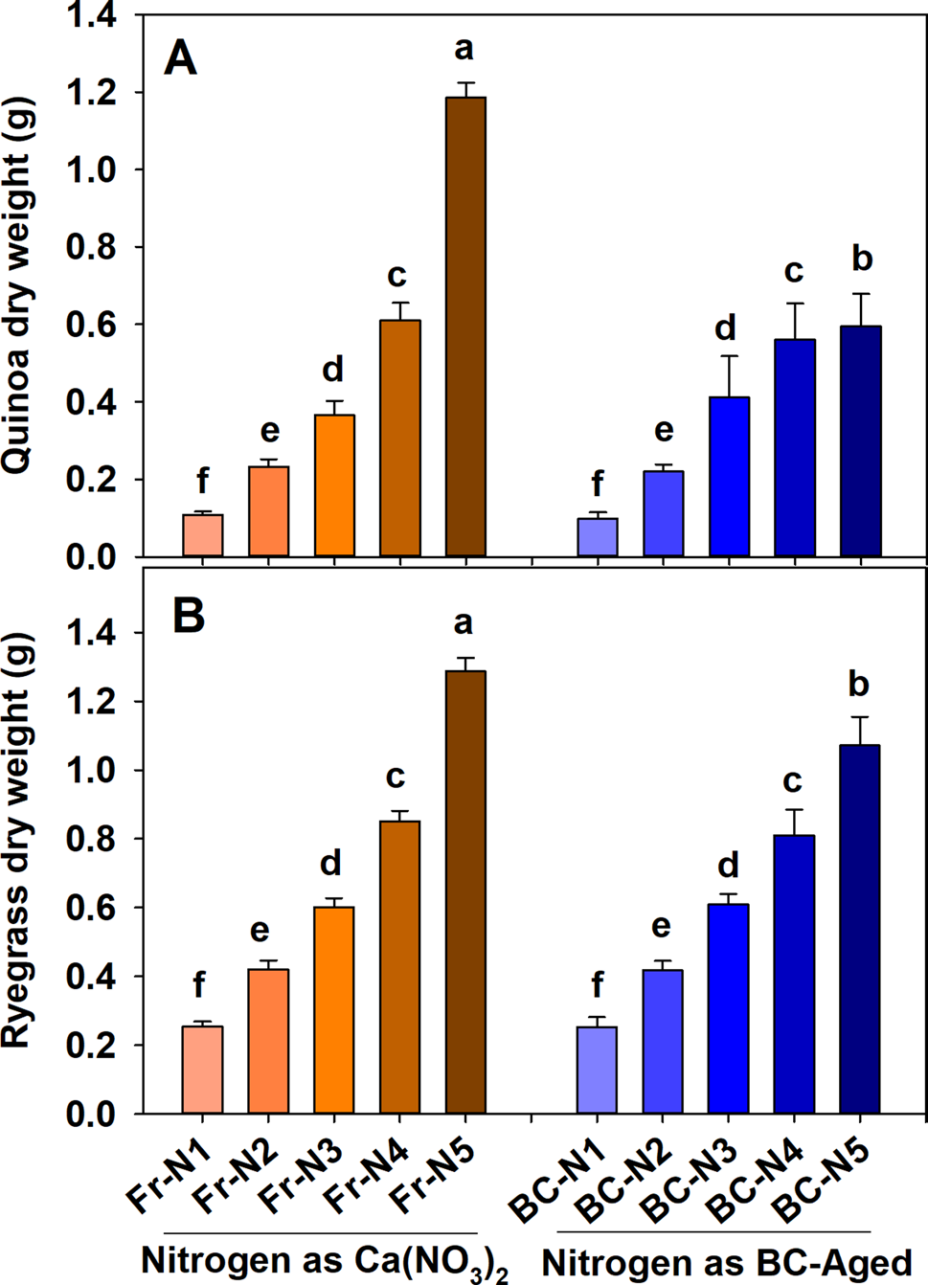
Effect of different nitrogen sources [(CaNO_3_)_2_ and N captured in BC-aged] and application levels (N-1 = 0, N-2 = 44, N-3 = 88, N-4 = 176 and N-5 = 352 kg ha^−1^) on quinoa and ryegrass dry matter production under controlled conditions (means + s.d., *n* = 4). The dry matter of ryegrass is the sum of two cuts harvested at 30 days interval. Note that the y-axis scales of (**A**) and (**B**) are different, based on the respective dry matter yield production from quinoa and ryegrass. Different letters on top of the bars indicate significant differences in biomass due to the respective N sources and N levels following two-way ANOVA (Tukey test, *P* < 0.05).

The dry matter yield of quinoa (Fig. 1A) and ryegrass (Fig. 1B) steadily increased with increasing rates of N addition with both N sources (synthetic fertilizer and N captured in BC-aged). However, in quinoa, at the highest application dose (N at 352 kg ha^−1^), the synthetic N source produced greater (*p* ≤ 0.001) dry matter yields (+ 99%), compared to N delivered from BC-aged. There was a greater (*p* ≤ 0.001) fresh to dry mass ratio between N sources at 352 kg ha^−1^, indicating a higher moisture content in the plant material in the BC-aged treatment (see supplementary information). Interestingly, up to the second-highest rate of application in quinoa (N at 176 kg ha^−1^), there was no significant difference in the aboveground biomass production between equal N supplies from the synthetic versus the BC-aged N sources (Fig. 1A). In ryegrass, N supplied via BC-aged also resulted in statistically similar dry matter yields compared to the synthetic N supply at all N levels except the highest amount (352 kg ha^−1^ N), where the synthetic N fertilizer again produced greater dry biomasses (+ 20%), compared to N applied as BC-aged.

### Effect of N sources on plant nitrogen uptake

**Figure 2:**
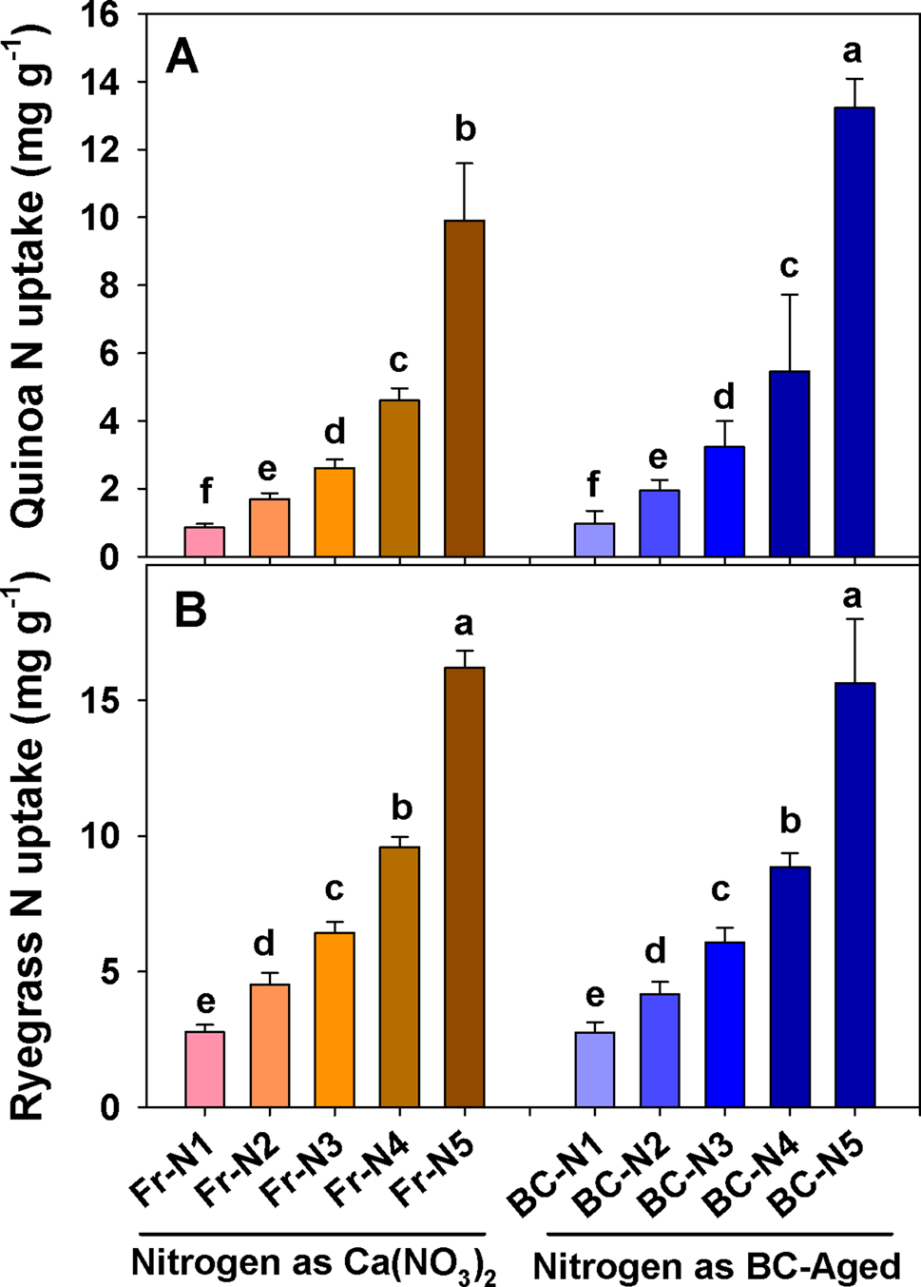
Effect of different nitrogen sources [(CaNO_3_)_2_ and N captured in BC-aged] and levels of N application (N-1 = 0, N-2 = 44, N-3 = 88, N-4 = 176 and N-5 = 352 kg ha^−1^) on N uptake in dry matter yield of quinoa and ryegrass (sum of two repeated cuttings at 30 days interval) under controlled conditions (means + s.d., *n* = 4). Note that the y-axis scales of (**A**) and (**B**) are different, based on the respective N uptake in dry matter yield of quinoa and ryegrass. Different letters on the bars indicate significant differences in the respective mineral N sources and levels following two-way ANOVA (Tukey test, *P* < 0.05).

Nitrogen uptake by quinoa and ryegrass from both N sources (mineral and BC-aged carrying N, mainly NO_3_^−^ and NH_4_^+^) increased with increasing rates of application (Fig. 2A,B). N concentrations in the quinoa tissue were significantly higher (58%) when the N was applied in the form of BC-aged, as compared to the mineral nitrate fertilizer at its highest rate of application (Table 1). The N uptake by aboveground plant biomass of quinoa per pot was up to 120% greater than that achieved with the application of mineral N at the highest N dose (352 kg N ha^−1^, Fig. 2A). In ryegrass, there was no significant difference in the tissue N concentration and hence in the total N uptake into the aboveground biomass between both N sources at any rate of application (Table 1, Fig. 2B).

**Table 1:** Plant (quinoa and ryegrass) tissue nitrogen concentration as influenced by nitrogen sources [(CaNO_3_)_2_ and BC-aged captured (NO_3_^−^ + NH_4_^+^)] at five rates of application (control (no nitrogen), 44, 88, 176 and 352 kg N ha^−1^), *n* = 4 per treatment.

Treatment factors	Quinoa N conc. (%)	Ryegrass N conc. (%)
**Nitrogen as (CaNO**_**3**_**)**_**2**_		
Control	0.81 ± 0.13	2.15 ± 0.13
44 kg N ha^−1^	0.73 ± 0.10	1.98 ± 0.02
88 kg N ha^−1^	0.72 ± 0.08	1.97 ± 0.06
176 kg N ha^−1^	0.76 ± 0.09	2.03 ± 0.04
352 kg N ha^−1^	0.83 ± 0.10	2.28 ± 0.06
Overall mean ± s.d	0.77 ± 0.04	2.08 ± 0.12
**Nitrogen as BC-aged captured (NO**_**3**_^**−**^ **+ NH**_**4**_^+^)		
Control	1.14 ± 0.45	2.15 ± 0.18
44 kg N ha^−1^	0.89 ± 0.12	1.89 ± 0.03
88 kg N ha^−1^	0.81 ± 0.10	1.88 ± 0.09
176 kg N ha^−1^	1.42 ± 0.41	2.03 ± 0.15
352 kg N ha^−1^	1.84 ± 1.00	2.76 ± 0.14
Overall mean ± s.d	1.22 ± 0.38	2.14 ± 0.32

The values are means and standard deviation.

### Mineral soil nitrogen (NO_3_^−^ and NH_4_^+^)

**Figure 3:**
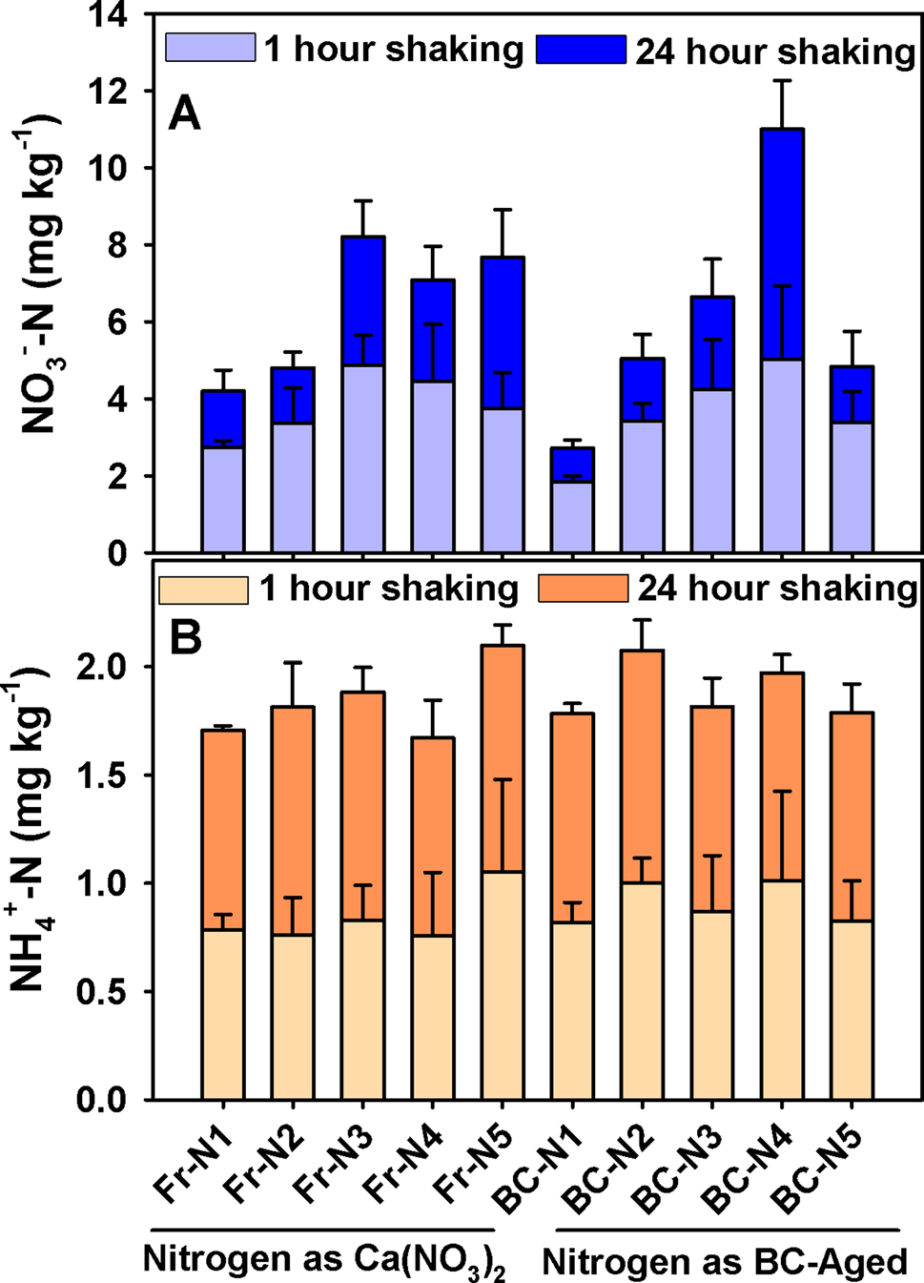
Effect of different nitrogen sources as [(CaNO_3_)_2_ and N captured in BC-aged] and levels of application (N-1 = 0, N-2 = 44, N-3 = 88, N-4 = 176 and N-5 = 352 kg ha^−1^) on leftover mineral N in potted soil after the harvest of quinoa under controlled conditions (means + s.d., *n* = 4). The mineral contents presented in the stacked bars are the sums of 1 + 24-h extractions (base and top respectively). Note that the y-axis scales of (**A**) (NO_3_^−^) and (**B**) (NH_4_^+^) are different, based on the respective N concentration in potted soil. There was no significant difference among N sources or levels following two-way ANOVA (*P* ≤ 0.05).

It has been shown earlier that the N (mostly NO_3_^−^) captured by biochar particles during its field aging was not easily extractable by standard methods, nor readily plant-available under field conditions^27,28^. Therefore, the remaining N from both sources of N application was assessed by repeated extractions. No significant difference in the form of remaining N (NO_3_^−^ or NH_4_^+^) was found between the two sources of N in the soil mixtures used in the study (Fig. 3A,B); overall, the remaining amounts were small.

### Properties of fresh and field-aged biochar

The characterization of functional groups on the surface of fresh and the BC-aged (crushed) is presented in Table 2. The fresh biochar had a higher concentration of carbon (both aromatic and nonaromatic) measured at 284.8 eV and 290.7 eV compared to the field-aged biochar. The BC-aged had a higher concentration of carboxylic and ketonic functional groups but a lower concentration of shakeup peaks that are indicative of aromatic carbon content. Nitrogen functional groups on the fresh biochar were associated with the carbon matrix, whereas on the field-aged biochar, N functional groups were mainly associated with sorbed nitrates and amino acids. The surface content of the N in the field-aged sample was approximately 3 times that of the fresh biochar. The Ca, Mg, S, and Cl on the surface also had higher concentrations.

**Table 2:** Region and survey XPS analysis of crushed fresh and field aged biochar.

**Peak table**							
Fresh-BC				BC-aged			
Name	Groups	Peak BE (eV)	Atomic %	Name	Groups	Peak BE (eV)	Atomic %
C 1*s* A	C–C/C––H	284.82	71.9	C 1*s* A	C–C/C–H	284.82	46.46
C 1*s* B	C–O/C–OC	286.42	10.09	C 1*s* B	C–O/C–OC	286.42	11.46
C 1*s* C	C=O/C–N	287.82	3.19	C 1*s* C	C=O/C–N	287.62	5.89
C 1*s* D	O=C–O	289.02	2.88	C 1*s* D	O=C–O	289.02	4.95
C1*s* E	Shakeup peak	290.69	2.48	C 1*s* E	Shakeup peak	290.7	1.5
C 1*s* F	Quinone	284	1.08	C 1*s* F	Quinone	283.5	0.27
N 1*s* A	Quaternary N (pyridine/neutral imine)	401.07	0.44	N 1*s* A	N–C–COOH /Pyridone	400.75	1.99
N 1*s* B		398.03	0.26	N 1*s* B	Nitrates	407.64	0.77
O 1*s*		532.79	7.67	O 1*s*		533.46	26.07
				S 2*p*		171.06	0.64

**Peak table survey**							
Name		Peak BE	Atomic %	Name		Peak BE	Atomic %
Si 2*p*		103.43	0.49	Al 2*p*		76.56	1.14
C 1*s*		284.88	90.35	Cl 2*p*		199.91	0.13
N 1*s*		401	0.82	C 1*s*		285.15	69.07
Ca		440.86	0.55	N 1*s*		400.88	2.02
O 1*s*		532.61	7.58	Ca 2*s*		440.96	1.48
Mg 1*s*		1,304.48	0.22	O 1*s*		533.5	25.4
				S 2*p*		171.06	0.64
				Mg 1*s*		1,305.18	0.45

### Organic carbon and nitrogen fractions

The fresh biochar had a slightly higher concentration of dissolved organic carbon (DOC) than the BC-aged and the highest DOC concentration was measured in fresh biochar in the 2nd extraction (Table 3). It suggests that volatile organic compounds were condensed during pyrolysis into the submicron pores. The relatively high content of DOC in the first extraction of the BC-aged indicated that these organic compounds have been adsorbed later from the soil onto the surface of the biochar. There was only a small concentration of hydrophobic organic compounds (HOC) although more were measured in the fresh biochar in the 2nd extraction. The BC-aged had a higher concentration of biopolymers and humic-like substances overall and these were measured in the first and second extractions (53.5% and 53.6% of the totals, respectively). The fresh biochar contained greater low molecular weight (LMW) neutrals than the BC-aged in the second extraction. The fresh biochar had a higher concentration of building blocks (polyphenols/polyaromatic acids) and LMW acids which are compounds that would have formed during pyrolysis at high temperatures.

**Table 3:** The concentrations of different DOC fractions extracted from fresh and field-aged biochar in three repeated extractions (1Et–3Et). *DOC* dissolved organic carbon, *HOC* hydrophobic organic carbon, *CDOC* chromatographable organic carbon, Building blocks are polyphenols/polyaromatic acids, *LMW neutrals* low molecular weight neutrals, *n.q.* indicate not quantified.

mg L^−1^	Fresh-BC (1Et)	Fresh-BC (2Et)	Fresh-BC (3Et)	BC-aged (1Et)	BC-aged (2Et)	BC-aged (3Et)
DOC	17.2	390.3	67.3	170.15	52.74	178.3
HOC	0.01	43.6	7.4	25.26	n.q	18.18
CDOC	17.2	346.7	59.9	144.9	52.74	160.11
Biopolymers	n.q	6.87	0.9	28.0	7.6	4.6
DON	0.1	n.q	n.q	n.q	n.q	n.q
N/C	15.2	n.q	n.q	n.q	n.q	n.q
Humics	1.8	34.5	8.5	91.0	28.28	29.7
DON	3	7.08	1.8	3.85	1.3	2.9
N/C	n.q	0.21	021	0.04	0.05	0.1
Building blocks	2.0	30.5	4.0	10.3	6.0	8.3
LMW neutrals	1.9	274.7	46.5	15.55	10.8	117.4
LMW acids	11.45	n.q	n.q	n.q	n.q	n.q

The BC-aged had a much higher concentration of dissolved nitrogen [sum of dissolved organic nitrogen (DON) and dissolved inorganic nitrogen (DIN)] and most of this nitrogen was released in the first extraction (Table 4). The highest DON of the fresh biochar was measured in the second extraction indicating that the N was held more tightly in the pores than in the BC-aged. Some of the DON was identified with the humic substances, with the highest concentration in the first extraction from the BC-aged and the second extraction from the fresh biochar.

**Table 4:** The concentration of dissolved organic nitrogen in fresh and field aged biochar.

Fresh-BC	Total dissolved N (DON) (mg L^−1^)	BC-aged	Total dissolved N (DON) (mg L^−1^)
(1Et)	1.267	(1Et)	312.66
(2Et)	247.84	(2Et)	162.67
(3Et)	32.77	(3Et)	123.65

### Surface structure of fresh and field-aged biochar

**Figure 4:**
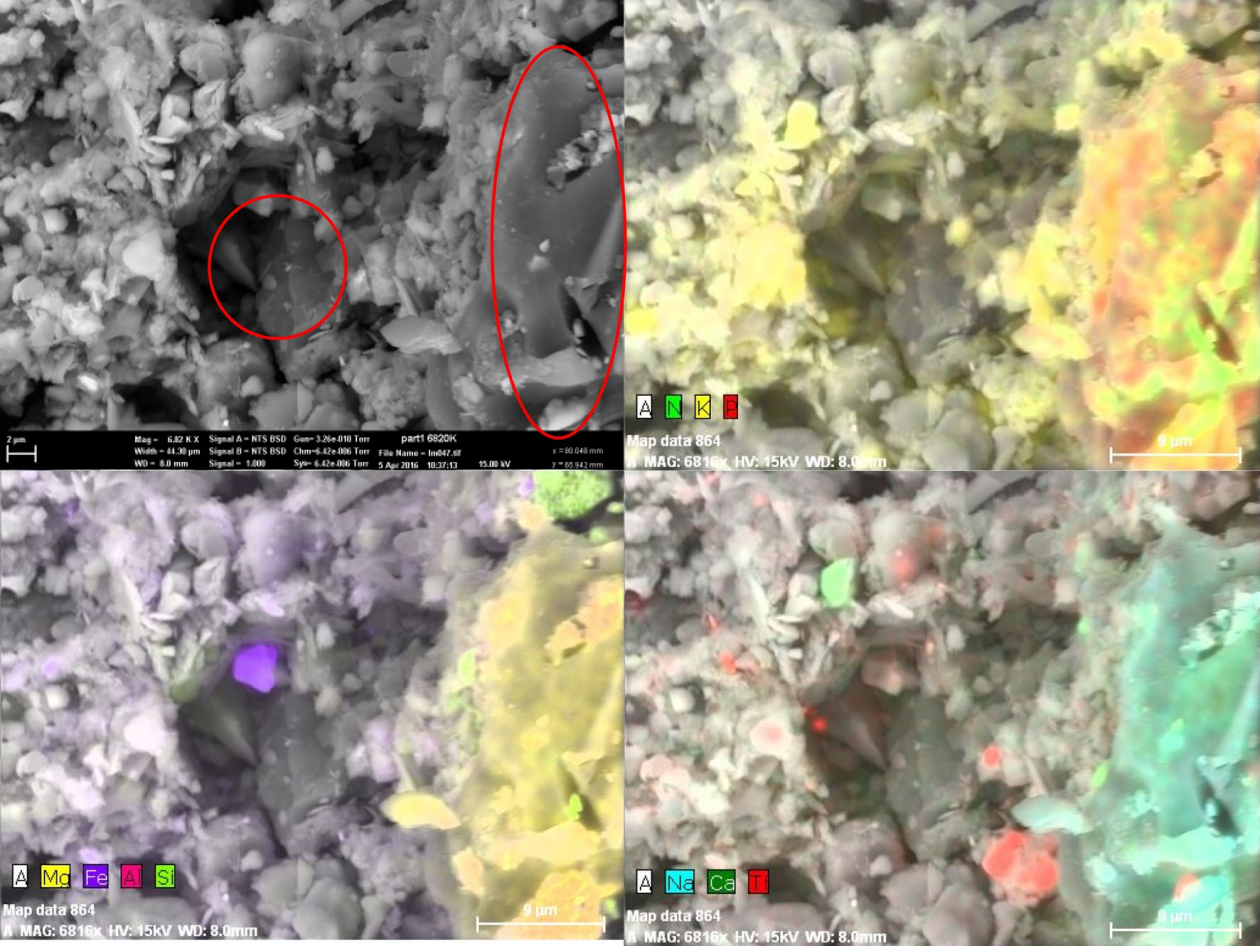
Secondary electron image of the surface of the aged biochar and EDS maps of this surface. Red circles indicate a biochar matrix that has a minerals’ coating on its internal pores and external surfaces.

The surface of the fresh biochar has a relatively high concentration of micron and submicron mineral particles embedded in the carbon matrix (Figure S3). The minerals on the surface of the pores could be coated in organic compounds that were deposited during pyrolysis (Figure S4). This would agree with the findings of the LC–OCD analysis of the extracts. Red circles of the secondary electron image of the surface of the BC-aged and EDS maps indicate that the biochar matrix has a minerals’ coating in its internal pores and external surfaces (Fig. 4).

**Figure 5:**
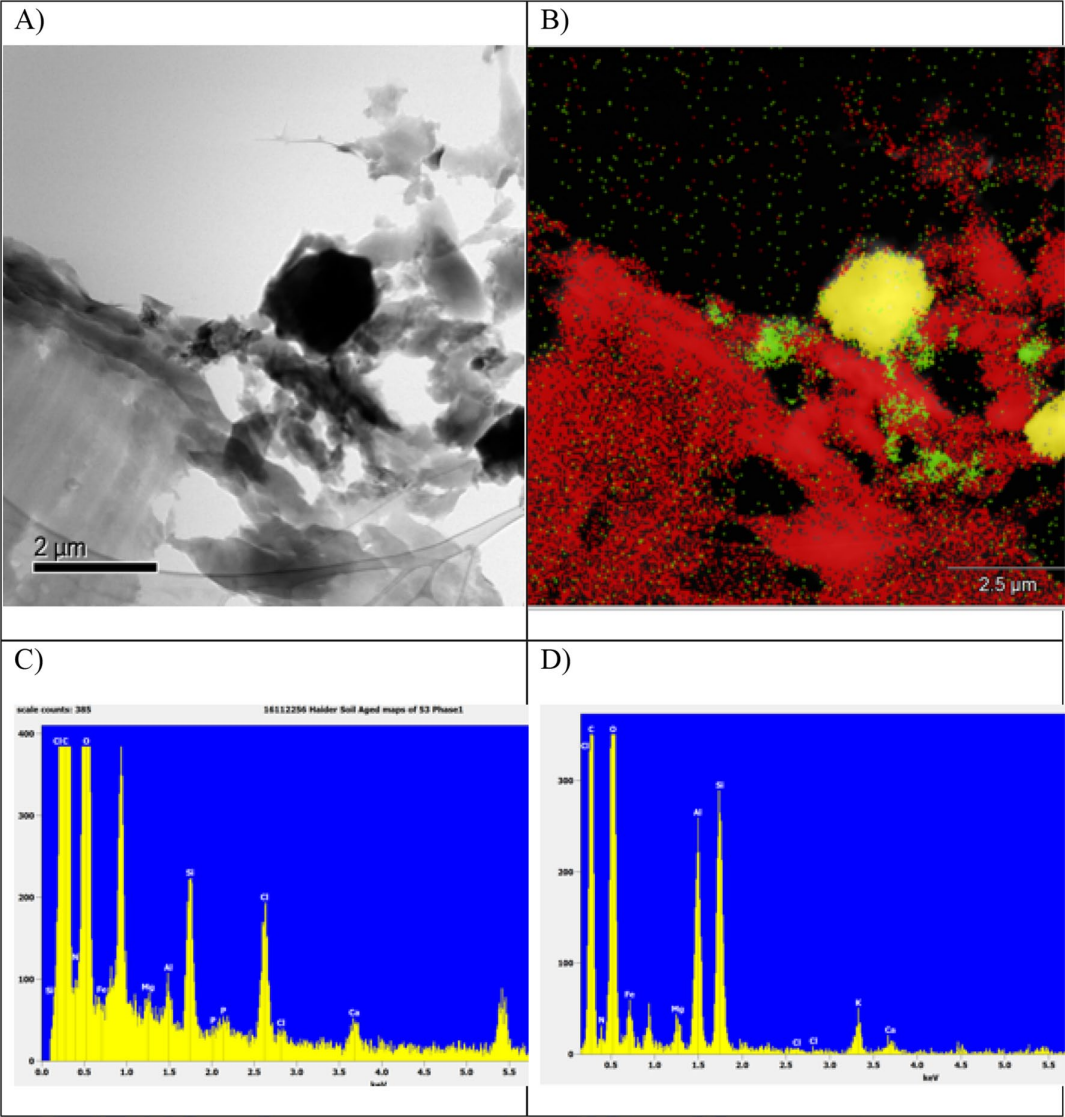
Image (**A**) is the bright field image of the surface of the aged biochar; (**B**) is the EDS phase map of image **A** (where red is a high carbon phase and yellow is a high silicon phase; (**C**) is the EDS map of red phase showing that minerals containing Si, Cl, Ca, Fe, Mg, and Al are associated with the carbon matrix; (**D**) is the EDS spectrum showing that the N is associated with mineral high in Al, Si, and Fe (probably clay).

There is a range of micron and submicron minerals that have been deposited on the surface during biochar ageing (Fig. 5). The areas where there are detectable levels of N are associated with the carbon matrix. A range of other elements (K, Na, P, and Ca) is also associated with this carbon matrix. High magnification imaging and elemental analysis with scanning transmission microscopy confirms the SEM observations. The detectable level of N is associated with submicron mineral phases that exist on the surface of the high carbon matrix of the biochar. These areas have a high macroporosity and therefore the N is accessible to both the plant root hairs and micro-organisms (Fig. 5).

## Discussion

This study aimed to test for the first time if N (largely nitrate) captured in field-aged biochar particles would be bioavailable to plants under controlled conditions. Based on earlier field results^28^ we hypothesized that only part of the captured N would be accessible by growing plants. Moreover, we expected that a higher percentage of biochar-N would be taken up by plants when N was a limiting resource (lower application rates), triggering root growth and foraging for N (which is a typical finding in biochar-amended soils, e.g.^33,34^), and a lower fraction when N was applied at larger doses.

The background of the study is the shifting paradigm of using biochar as a carbon compound/carrier material for developing carbon fertilizers instead of applying large amounts, production-fresh and untreated, to soils^23,30,35^. The use of comparably large amounts of biochar in this study (used as “biochar-nitrate/N”) does not contradict this aim, because it is in line with recent studies of concentrated use of biochar in the root zone (conservation farming approach^36^) with locally high dosages, but overall as low as 2–4 t ha^−1^. Recently, Schmidt et al*.*^37^ reported that the use of biochar–urine mixtures, placed with compost concentrated in the root zone at dosages of 1–2 t ha^−1^, produced on average double yields in 21 field trials in fertile soils in Nepal (with a monsoon climate, i.e. usually probably with N leaching losses). One of the open questions was if the temporary uptake and re-delivery of N from biochar particles could mechanistically explain part of the success of this novel approach, i.e. if low-molecular mineral or organic N captured by biochar would be plant-available, and to what degree.

Our results reveal that the captured N in field-aged biochar was nearly completely plant available to both, perennial ryegrass and quinoa under controlled conditions, which essentially contradicts our initial hypotheses that part of the N would remain biochar-bound, and that the amount that was retrieved by plants would depend on the N dosage.

These results raise the question of why the captured N in the field (in aged biochar particles)^28^ was less plant available in the field study than in this pot study. We assume that in contrast to this study, N-carrying biochar particles were spread widely in the field soil when plowed in homogenously in 2012, with the plant roots having to grow long distances towards the nearest N containing biochar microsites. In contrast, in this 200 g soil + biochar mixture in a pot (which was more similar to a concentrated root zone application), roots were in close proximity to biochar particles (see Supplementary Figure S1, washed root system with clinging biochar particles). The difference between the field and pot study results highlight that (1) roots may not per se be growing towards nutrient-loaded biochar particles, or that (2) there may be a minimum distance between root tips/mycorrhizae and biochar-N reservoirs that must be met for effective root uptake. Principally, root growth, mycorrhization, and nodulation (legumes) all respond positively to (pure) biochar application, as indicated by meta-analysis^34^. Also, Hammer et al*.*^38^ showed that mycorrhizae were able to mine P from loaded biochar particles in a controlled model setting without plants.

Our study is in line with results by Kammann et al*.*^25^, also using quinoa, that plants can utilize N (nitrate) from naturally loaded (aged/coated) biochar particles. The combined observations of the field study and this pot study, however, suggest that a more concentrated root zone placement together with the nutrients (and potentially clay minerals if available, see Qian et al*.*^35^) may be favorable in terms of N capture and release to crop plants, as described by Schmidt et al*.*^37^.

### The impact of N levels and sources of application on plant growth and N uptake

The plants’ fresh and dry biomass yield was statistically similar up to 176 kg N ha^−1^ (i.e. near the optimum N requirements of quinoa and ryegrass), while the highest (352 kg N ha^−1^) N application with field-aged-BC did not further improve yields, posing the question if upper limits exist of beneficial biochar concentrations in the plant root zone.

Interestingly, at this N fertilization rate, the total N uptake by quinoa was significantly higher (120%) when the N was supplied from BC-aged, compared to the synthetic N application; the latter, however, showed untypical morphology (Figure S1). Soil mineral N analysis after quinoa harvest revealed no significant difference among the treatments, suggesting that quinoa plants successfully mined the biochar particles (also in the highest N treatment) for the captured N, despite their growth anomaly. Since all other nutrients were additionally supplied, one potential explanation might be the sorption of essential micronutrients such as boron, copper or zinc to the surfaces of the biochar particles at the highest N application dose as observed by Zemanová et al*.*^39^. In general, quinoa has a high N uptake efficiency^40^. However, in a study with high and low N supply to different quinoa genotypes, Bascuñán-Godoy et al*.*^41^ observed 25% less carbon assimilation, smaller and thinner leaves by some of the tested genotypes with high N application, which may explain the growth anomaly observed in the present study at the highest N application dose via BC-aged (Figure S1). This may also suggest that the BC-captured N must have been easily extractable from the particles by the quinoa roots despite the shoot morphological anomaly.

The biochar amount of 6.03 g (at 352 kg N ha^−1^) would equal an application dose of 33.92 t ha^−1^ of biochar (when applied homogeneously and plowed in) and it might be argued that such a large biochar dosage may change the soil’s pH value, impacting micronutrient availability. However, since the pH of the aged biochar was even lower than that of the field soil (6.0 and 6.31 for BC-aged and field soil, respectively), the biochar addition is unlikely to have impacted (micro) nutrient availability via pH changes.

With *Lolium perenne*, no significant difference in the total N uptake or tissue N concentration was observed with both N sources at any rate of application, suggesting that ryegrass roots were able to successfully mine biochar particles for the captured N and mobilize/drain it from the particles.

The question is why the nitrate has nearly completely been drained from the biochar particles in this study, while this did not occur in the field study of Haider et al*.*^28^. We hypothesize that the accessibility of biochar-captured nitrate or N, in general, may, in addition to the distance between roots and biochar particles (i.e. application mode), largely depend on the soil water availability; and with the ion uptake gradient that a root-biochar contact is able create. Classical chemical nitrate sorption/desorption processes of nitrate from aged, organically coated biochars may play a small role^29,30^, but chemically aged (oxidized) biochars can lose some of their (already small) anion exchange capacity^42^. Hence, the main mechanism of nitrate capture is unlikely to be chemical sorption to biochar surfaces per se. Hence, the release pathway (and delivery to plant roots) also needs to be different. Previous studies showed that repeated extraction cycles and/or longer extraction times increased the amount of nitrate that could be retrieved from N-enriched, aged or co-composted particles^25,27,31^. Thus, the constant water supply of 60–65% WHC in the pots, together with the root proximity to the particles, may have provided ideal conditions for nitrate extraction by plant roots. We hypothesize that plant roots will create a concentration gradient between a higher nitrate concentration in the biochar pores and a lower concentration at the root surface where nitrate is taken up, thus draining pores over time, given that enough water is present to allow nitrate diffusion in (water filled) pores^43^. The main factors determining the movement of nitrate during pore drainage (by plant N uptake) may hence be (1) diffusion along this gradient, (2) the biochar properties that determine surface (water) interactions, and (3) the clogging of the pores by minerals or organic molecules. Biochar properties include (1) pore size distribution (initial and after aging), (2) surface properties such as induced dipole forces of the biochar-coating combination (by charge-transfer interactions, water bridging, and van der Waals interactions^44^) or (3) general water movement characteristics on inner biochar (pore) surfaces^43^. While Joseph et al*.*^29^ assumed a blocking of biochar pores with organo-mineral layers during composting, essentially trapping nutrients inside biochar pores, Hagemann et al*.*^30^ showed that the organo-mineral coating itself (which formed on biochar particles during composting) was highly enriched in N.

Regardless of the mechanism of capture or exact location of nitrate capture, our study demonstrates for the first time that the biochar-captured nitrate was largely extractable by plant roots. This may aid the development of biochar-based fertilization techniques or for buffering N fluctuations for crops in soils, as long as the soil moisture conditions allow nutrient flow from biochar particles to plant roots. However, the soil water potential (pore size distribution of the soil–biochar continuum) may limit the extractability. We hypothesize that moisture/soil suction thresholds will exist where nitrate-enriched biochar particles can no longer be mined by plant roots, which should be tested in future studies.

## Materials and methods

### Characterization of experimental soil and site description

The soil used in this experiment was collected from the plow layer of an experimental site at the Research Station of the Institute for Plant Breeding and Agronomy I, Justus Liebig University Giessen, Germany. The Research Station is located at 49° 45′ N and 8° 29′ E, 90–145 m (above sea level) at Gross-Gerau in the federal state of Hesse. The sandy soil at the experimental station was formed from the sand deposits of the river Rhine flowing in the west of the experimental site. The river Main is flowing in the North and, the eastern side of the experimental area is surrounded by the Odenwald Mountains. The site is rain-fed but frequently irrigated with sprinkler irrigation in case of longer drought periods during the summers. The average (56 years) precipitation and temperature of the region are 600 mm and 9.8 °C, respectively. The soil was characterized as silty sand, containing 85.2, 9.6, and 5.2% sand, silt, and clay respectively. The soil with a pH of (0.01 M CaCl_2_) 6.3 contains 0.59% total organic carbon, 0.06% total nitrogen, 7.42 mg kg^−1^ nitrate, and 0.531 mg kg^−1^ ammonium. The soil available phosphorous contents is 92.2 mg kg^−1^ (calcium-acetate–lactate (CAL) as the extractants), available potassium content is 124.5 mg kg^−1^ (CAL extractants), and Magnesium is 35.5 mg kg^−1^.

### Feedstock, fresh and field-aged biochar description

A feedstock mixture of two different tree species *Picea abies* L. and *Fagus sylvatica* L., (Norway spruce and European Beech, respectively) was used for biochar production. The overall feedstock was comprised of small wood chips, bark, twig pieces (70%), and needles (30%). Biochar was commercially produced by Pyreg GmbH, Dörth, Germany, at pyrolysis temperatures of 550–600 °C. The detailed chemical properties, particle size distribution, and elemental composition of BC are provided in Haider et al*.*^27,28^. Fresh BC was applied to the soil surface and incorporated to 15 cm depth in April 2012. The pre-existing crop rotation and conventional fertilizer regimes of maize (*Zea maize* L.), wheat (*Triticum aestivum* L.), barley with the intercrop pea (*Pisum sativum* L.) are described in Haider et al*.*^28^.

Biochar particles were retrieved at the end of the fourth major crop Maize in 2015 by sieving field soil samples after air-drying and picking particles from the sieved larger non-biochar detritus. At the time of application (spreading on experimental plots and then incorporating in 0–15 cm) biochar particle size fraction ranged > 6.3% to < 0.1% see details in Haider et al. 2015. Particles of 2–5 mm from field aged and laboratory-stored biochar (saved from the field-applied production lot (Fresh-BC), kept in an air-tight container) were ground to powder and analyzed for total carbon and nitrogen with a CN analyzer (Vario MAX, Elementar Analysensysteme Gmbh, Hanau, Germany). Fresh and field aged BC contained 0.55% and 0.98% N, respectively, where the fresh biochar naturally contains heterocyclic but no mineral N, whereas the aged biochar had additionally loaded itself with mineral N (see Table S1). Hence, for the nitrogen dosage, we analyzed BC-aged with an extended time of shaking and temperature^27^ and calculated with the maximum amount of 5,305.53 mg kg^−1^ (4,568.3 + 737.23, NO_3_^−^ and NH_4_^+^ respectively) mineral N contained in BC-aged, a conservative approach to the experimental set-up detailed below.

### Experimental setup

The experiment followed a completely randomized design (CRD) with two different plant species (quinoa and perennial ryegrass) in a controlled climate chamber at the Experimental Station of the Institute of Plant Nutrition, Justus Liebig University Giessen, Germany. The treatments for each crop were designed as follows: five levels of nitrogen supply (Control: no nitrogen addition), and 4, 8, 16, 32 mg N pot^−1^. Each pot was filled with 200 g ≤ 5 mm sieved sandy soil (taken from the experimental site described in Haider et al*.*^27,28^) + 50 g quartz sand to cover the seeds. The N application doses equalled 0, 44, 88, 176 and 352 kg N ha^−1^ (on soil weight basis with a bulk density of 1.5 kg L^−1^) obtained from two different sources (A) analytical grade calcium nitrate (Ca(NO_3_)_2_) by Sigma-Aldrich, and (B) from captured mineral N in BC-aged. The BC-aged required to meet mineral N doses (0, 4, 8 16 and 32 mg N per pot) was equivalent to 0, 4.22, 8.49, 16.99, and 33.92 Mg ha^−1^. Increasing rates of N application were selected to test, (a) what level of N, supplied by N-carrying aged biochar, would equal a certain amount of analytical grade mineral N and thereby, (b) to determine how much of the biochar-bound N may not be plant available. Each treatment pot was supplied and thoroughly mixed with a 7.0 mL of micronutrient solution containing the following nutrients per Litter: 0.86 g boron (H_3_BO_3_), 6.4 g copper (CuSO_4_ × 5 H_2_O), 0.06 g molybdenum (ammonium molybdate), 8.2 g manganese (MnSO_4_ × H_2_O), and 14.3 g zinc (ZnSO_4_ × 7 H_2_O). Ten seeds of quinoa, respective 0.35 g of ryegrass seeds were sown into each pot on Dec 18, 2015. After the emergence, only three well-growing quinoa plants of equal size per pot were maintained; perennial ryegrass seedlings were all maintained. The water holding capacity of the soil or soil–biochar mixture was determined beforehand by the procedure described by Kammann et al*.*^45^, and experimental pots were adjusted to 65% of the maximum water holding capacity every second day on weight loss basis. The pots (5 N levels × 2 nitrate “fertilizers” × 4 replicates, *n* = 40 for each crop), were installed in CRD with a factorial arrangement.

### Harvest and nitrogen uptake

Quinoa was harvested 38 days after sowing on January 26, 2016. Ryegrass was harvested twice, at 30th and 60th of sowing on January 18 and second on February 16, 2016, respectively. The total fresh mass of all plant parts (aboveground) per pot was recorded and plant biomass was oven-dried at 70 °C for 48 h. Total dry mass was noted, and plant samples were ball milled for nutrient analysis. Total carbon and nitrogen analyses of the plant material were performed with a CN analyzer (Vario MAX, Elementar Analysensysteme Gmbh, Hanau, Germany). Pot N uptake was calculated by multiplying the harvested biomass per pot with the N concentration in the plant material.

### Experimental soil extraction for mineral N after harvest

After the harvest of quinoa, the quartz sand at the top surface of each pot was removed and plant roots were gently recovered. Fresh soil samples were sieved (2 mm) and extracted twice for N_min_ (NO_3_^−^ and NH_4_^+^). During the first extraction, we followed the standard 2 M KCl extraction method^46^. A 20 g soil was weighed in 80 mL 2 M KCl (1:4 w/v soil/KCl), shaken for 1 h at 100 rpm, and filtered (round filter ø 70 mm S&S type 595). After the first extraction, the filter papers containing the soil and the biochar particles were placed in new extraction bottles and another 80 mL of fresh 2 M KCl solution was added. This time, samples were shaken in a hot-water bath (80 °C) for 24 h to extract potentially trapped nitrate from the biochar particles^27^. Again, the extractant was collected by filtering the extraction solution. The concentration of mineral N (NO_3_^−^ and NH_4_^+^) was quantified colorimetrically using an auto-analyzer (Seal, Germany). Unfortunately, we were unable to extract the soil after the ryegrass harvest due to difficulties to remove the very fine roots properly.

### Characterization of the fresh and the field-aged biochar

To determine where and what type of nitrogen is existing in the fresh and field-aged biochar samples were analyzed using X-ray Proton spectroscopy (XPS), FEI corporation NanoSEM 230 scanning electron microscope (SEM) and JOEL ARM transmission electron microscope. Details of the techniques and equipment are given in Joseph et al*.*^29^ and Archanjo et al*.*^47^.

To determination the dissolved organic carbon from biochar samples, 1 g of fresh and field-aged biochar samples were finely ground and added to 10 mL distilled water. The solutions were regularly stirred at 50 °C for 24 h and subsequently, filtered to separate the solid and liquid phases [first extraction (1Et)]. The pH value was adjusted before measuring the DOC content from the samples. Liquid Chromatography—Organic Carbon Detection (LC–OCD) was performed on the solutions and results were analyzed using customized software (ChromCALC, DOC-LABOR, Karlsruhe, Germany).

To measure the concentration of DOC in the second extraction (2Et), 10 mL distilled water was added to the solid phase obtained from the first extraction (residual). Likewise, 10 mL of distilled water was added to the solid phase from the second extraction (residual) to obtain third extraction (3Et). The concentration of TC/TN was also measured in each step using Multi N/C Analyser.

### Statistical analysis

Results were analyzed separately for both plant species (quinoa and perennial ryegrass) because of their different growth habits. We employed two-way ANOVA for both data sets by using SigmaPlot 13.0 (Systat, Inc., Richmond, USA) at a significance level of *P ≤ *0.05. Data were tested for normality and homogeneous variances with the Shapiro–Wilk test and Brown-Forsythe test, respectively. Where needed, data were transformed (simple statistical transformations in SigmaPlot 13.0) before statistical analysis. The Tukey’s Honest Significant Difference (HSD) test was carried out to identify treatment level effects such as N application dose or N source (biochar vs. mineral) at the individual treatment levels (*P ≤ *0.05).

## Supplementary information

Supplementary Information.

## Abbreviations

BC: Biochar
BC-aged: Field-aged biochar
