# Supplementary material for: Mineral nitrogen captured in field-aged biochar is plant-available
Source: Sci Rep. 2020 Aug 14;10:13816. doi: 10.1038/s41598-020-70586-x (PMC7427999; doi:10.1038/s41598-020-70586-x)
Supplement: Supplementary file 1 — Supplementary Information. [file 41598_2020_70586_MOESM1_ESM.docx]

**SUPPLEMENTARY INFORMATION**

**Title:** **Mineral nitrogen captured in field-aged biochar is plant-available**

Ghulam Haider^1,^*, Stephen Joseph^2,3^, Diedrich Steffens^4^, Christoph Müller^5,6^, Sarasadat Taherymoosavi^2^, David Mitchell^7^, and Claudia I. Kammann^8,^*

*^1^Department of Plant Biotechnology, Atta-Ur-Rahman School of Applied Biosciences, National University of Sciences and Technology. NUST Campus, H-12, Islamabad, Pakistan, Phone: +92 5190856142. E-mail: [ghulam.haider@asab.nust.edu.pk](mailto:ghulam.haider@asab.nust.edu.pk)

^2^School of Materials Science and Engineering, University of NSW., Kensington, NSW Australia 2052

^3^Discipline of Chemistry, University of Newcastle, Callaghan, NSW 2308, Australia

^4^Department of Plant Nutrition, Justus-Liebig University Giessen, Heinrich-Buff Ring 26-32, 35392 Giessen, Germany. Phone: +49 0641 99-39165. Fax No. +49 0641 99-39160. E-mail: [diedrich.steffens@ernaehrung.uni-giessen.de](mailto:diedrich.steffens@ernaehrung.uni-giessen.de)

^5^Institute of Plant Ecology, Justus-Liebig University Giessen, Heinrich-Buff Ring 26-32, 35392 Giessen, Germany. Phone: +49 641 99 35336. Fax No. +49 641 99 35309. E-mail: Christoph.Mueller@ucd.ie

^6^School of Biology and Environmental Science and Earth Institute, University College Dublin, Belfield, Dublin 4, Ireland

^7^Electron Microscopy Centre, AIIM Building, Innovation Campus, University of Wollongong, Squires Way, North Wollongong, NSW 2517, Australia

*^8^Climate Change Research for Special Crops, Department of Applied Ecology, Hochschule Geisenheim University, Von-Lade Str. 1, D-65366 Geisenheim, Germany. Phone: +49 6722 502 755 or +49 171 3531553, Fax No. +49 6722 502 430. E-mail: [Claudia.Kammann@hs-gm.de](mailto:Claudia.Kammann@hs-gm.de)

*Corresponding Author(s): [ghulam.haider@asab.nust.edu.pk](mailto:ghulam.haider@asab.nust.edu.pk) ; [Claudia.Kammann@hs-gm.de](mailto:Claudia.Kammann@hs-gm.de)


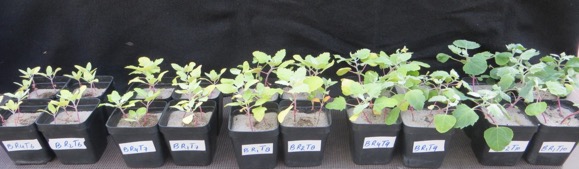

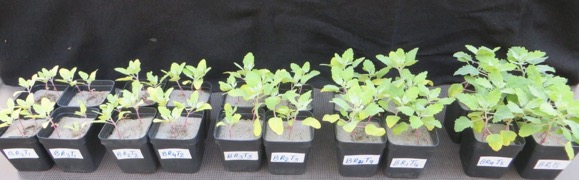

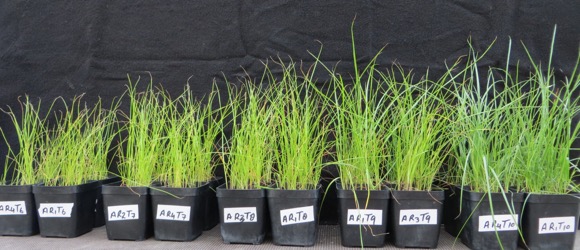

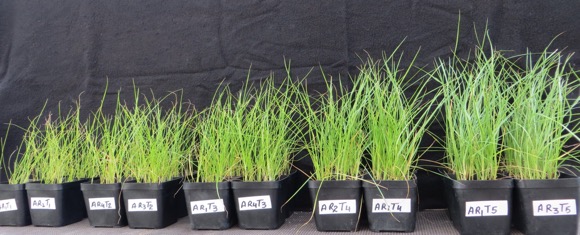


**Control**

**44 kg N ha^-1^**

**88 kg N ha^-1^**

**176 kg N ha^-1^**

**352 kg N ha^-1^**

**Control**

**44 kg N ha^-1^**

**88 kg N ha^-1^**

**176 kg N ha^-1^**

**352 kg N ha^-1^**

**N as (CaNO_3_)_2_**

**N as BC-aged captured (NO_3_^-^ + NH_4_^+^)**

**N as (CaNO_3_)_2_**

**N as BC-aged captured (NO_3_^-^ + NH_4_^+^)**

**Figure S1.** The pictures show the experimental cultures quinoa and ryegrass. It is clearly visible that plant growth was increasing with increasing rates of N from both, either synthetic fertilizer (as (CaNO_3_)_2_), or as BC-aged, where N (mostly nitrate) was captured on in biochar particles during field ageing.


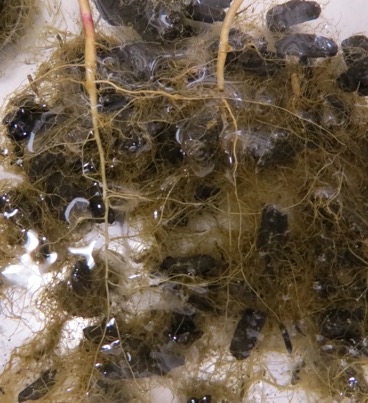

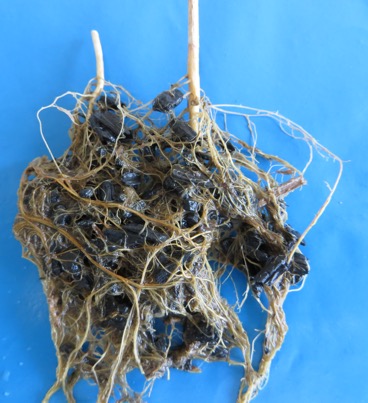


**Figure S2**. The photos shows the root system of quinoa plants after harvest and root washing of two pots of the 352 kg N ha^-1^ treatment. It is clearly visible that the roots form a close association with biochar particles that even withstands washing.


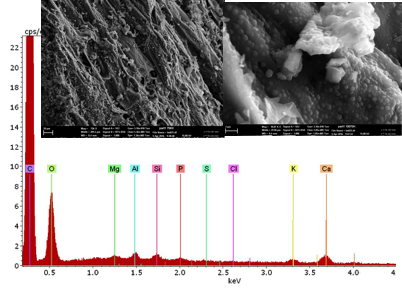


**Figure S3.** Secondary electron image and EDS analysis of fresh biochar

**Figure S4.** Secondary electron images of the pores and surfaces of the biochar.

**Table S1.** Carbon and nitrogen contents of fresh (saved in an airtight container in the laboratory) and field-aged biochar particles (≥2 to 5 mm), respective re-picked from a field experiment in a temperate sandy soil (methods and field experiment: see Haider et al. 2016, 2017).

| **Parameter** | **Fresh Biochar** | **BC-aged (field)** |
| --- | --- | --- |
| **C (%)** | 78.72 | 65.93 |
| **N (%)** | 0.55 | 0.98 |
| **NO_3_^-^ (mg kg^-1^)** | 8.1 | 4568.3 |
| **NH_4_^+^ (mg kg^-1^)** | 1.17 | 737.3 |
| **pH** | 9.0 | 6.0 |
